# Comprehensive investigation of malignant epithelial cell-related genes in clear cell renal cell carcinoma: development of a prognostic signature and exploration of tumor microenvironment interactions

**DOI:** 10.1186/s12967-024-05426-x

**Published:** 2024-07-01

**Authors:** Songyang Liu, Ge Li, Xiaomao Yin, Yihan Zhou, Dongmei Luo, Zhenggang Yang, Jin Zhang, Jianfeng Wang

**Affiliations:** 1 Department of Urology, Ren Ji Hospital, School of Medicine, Shanghai Jiao Tong University, Shanghai, China; 2 Department of Gastrointestinal Surgery, Tongji Hospital, School of Medicine, Tongji University, Shanghai, China; 3 Department of Internal Medicine, Shanghai Gongli Hospital, Second Military Medical University, Shanghai, China

**Keywords:** Clear cell renal cell carcinoma, Machine learning, Prognosis, Single-cell RNA sequencing, Cell communication

## Abstract

Clear cell renal cell carcinoma (ccRCC) is a prevalent malignancy with complex heterogeneity within epithelial cells, which plays a crucial role in tumor progression and immune regulation. Yet, the clinical importance of the malignant epithelial cell-related genes (MECRGs) in ccRCC remains insufficiently understood. This research aims to undertake a comprehensive investigation into the functions and clinical relevance of malignant epithelial cell-related genes in ccRCC, providing valuable understanding of the molecular mechanisms and offering potential targets for treatment strategies. Using data from single-cell sequencing, we successfully identified 219 MECRGs and established a prognostic model MECRGS (MECRGs’ signature) by synergistically analyzing 101 machine-learning models using 10 different algorithms. Remarkably, the MECRGS demonstrated superior predictive performance compared to traditional clinical features and 92 previously published signatures across six cohorts, showcasing its independence and accuracy. Upon stratifying patients into high- and low-MECRGS subgroups using the specified cut-off threshold, we noted that patients with elevated MECRGS scores displayed characteristics of an immune suppressive tumor microenvironment (TME) and showed worse outcomes after immunotherapy. Additionally, we discovered a distinct ccRCC tumor cell subtype characterized by the high expressions of *PLOD2* (procollagen-lysine,2-oxoglutarate 5-dioxygenase 2) and *SAA1* (Serum Amyloid A1), which we further validated in the Renji tissue microarray (TMA) cohort. Lastly, ‘Cellchat’ revealed potential crosstalk patterns between these cells and other cell types, indicating their potential role in recruiting CD163 + macrophages and regulatory T cells (Tregs), thereby establishing an immunosuppressive TME. PLOD2 + SAA1 + cancer cells with intricate crosstalk patterns indeed show promise for potential therapeutic interventions.

**Supplementary Information:**

The online version contains supplementary material available at 10.1186/s12967-024-05426-x.

## Introduction

Renal cell carcinoma (RCC) is a common malignant tumor of the kidney, originating from renal epithelial cells. Clear cell renal cell carcinoma (ccRCC), the most prevalent histological subtype, accounts for approximately 75–80% of cases and its global incidence is on the rise [1, 2]. Approximately one-third of RCC patients are diagnosed with distant metastasis initially, and the 5-year overall survival rate for these individuals is approximately 14% [1]. Certainly, incorporating novel nanomaterials into the treatment of cancers holds great promise for improving patient outcomes [3–5]. Currently, although there are multiple targeted drugs available, surgical resection is still the preferred treatment method for ccRCC [6]. However, it is disappointing that even with surgical resection, ccRCC still has a high recurrence rate. Approximately one-third of patients will experience tumor recurrence or metastasis after surgery [7]. Additionally, ccRCC has limited response to radiation therapy and chemotherapy, which further limits treatment options [8]. Targeted therapies, such as tyrosine kinase inhibitors (TKIs) and mammalian target of rapamycin (mTOR) protein inhibitors like everolimus, are the main adjuvant treatment strategies for ccRCC patients’ post-surgery [9, 10]. In recent years, immune checkpoint inhibitors (ICIs) have shown promising potential as a treatment method for various types of cancers [11]. By eradicating tumor cells and reversing the exhaustion of T cells, ICI therapy is now the established standard immunotherapy for advanced RCC. However, only a proportion of patients have attained notable and enduring advantages [12, 13]. Hence, there is an increasing demand for novel biomarkers or signatures that can accurately predict prognosis and guide treatment decisions within the realm of precision medicine, ultimately enhancing ccRCC patient outcomes.

To improve the treatment of ccRCC, deep understanding of the intricate and complex interplay between renal cancer cells and the tumor microenvironment (TME) is required [14]. This interaction is crucial for tumor evolution, invasion, metastasis, and response to therapy [15, 16]. The TME includes various components such as immune cells, fibroblasts, blood vessels, and extracellular matrix [17]. Advancements in single-cell RNA sequencing (scRNA-seq) technologies have rapidly enhanced the identification of various cellular populations and phenotypic states within tumors and provided comprehensive understanding of tumor heterogeneity and evolution [18].

In this study, by analyzing scRNA-seq data and bulk RNA sequencing data, the malignant epithelial cell-related gene signature (MECRGS) was developed based on 101 machine learning algorithms. The performance of the MECRGS in predicting prognosis, immunotherapy response, and its relation with immune and clinical characteristics in ccRCC patients was systematically explored. *PLOD2* (procollagen-lysine,2-oxoglutarate 5-dioxygenase 2), *C1S* (Complement component 1s), *C1R* (Complement component 1r), model genes of MECRGS, were associated with poor prognosis and particularly prominent in the most malignant epithelial cluster with high MECRGS scores, CNV scores, and stemness scores. The PLOD2 + SAA1 + tumor subtype displays intricate intercellular crosstalk and provides insights into the underlying molecular pathogenesis for ccRCC.

## Materials and methods

### Sources and preprocessing of datasets

This research utilized a total of 6 independent public bulk ccRCC RNA datasets obtained from various repositories, including TCGA-KIRC (https://portal.gdc.cancer.gov/), CPTAC (https://pdc.cancer.gov/pdc/), E-MTAB-3267, E-MTAB-1980 (https://www.ebi.ac.uk/arrayexpress/), GSE22541, and GSE29609 (http://www.ncbi.nlm.nih.gov/geo/), to develop and validate our model. The GSE53757 dataset with relevant clinical information was downloaded for validation. To elaborate, the GSE53757 dataset lacks survival time and status information, which made it unsuitable for survival analysis. However, it was instrumental in investigating the relationship between patients’ clinical stage and our signature (Supplementary Fig. 2T). The TCGA-KIRC dataset was utilized as the training dataset, with the other datasets serving as validation sets. The expression levels were initially converted from counts to TPM (transcripts per million), and then a log2 transformation of (TPM + 1) was applied. In the gene microarray data, the expression levels were subjected to RMA background correction and subsequently log2 transformed.

### Single‑cell RNA‑seq data collection and analysis

The scRNA-seq datasets including ccRCC tissues and benign kidney tissues were obtained from the Figshare-GX [19] and GEO database under the accession number GSE159115, GSE210042, GSE178481, and GSE156632. To ensure data quality, we performed initial quality control by preserving cells with mitochondrial gene content of less than 20% and genes expressed in at least three cells within a specific expression range (200 to 7000). These quality control steps are aimed at excluding potential low-quality cells, doublets, or other factors that may affect the accuracy of downstream analysis [20]. Subsequently, we identified highly variable genes, selecting 2000 of them for principal component analysis (PCA) to reduce dimensionality. We then utilized the “Harmony” package to mitigate batch effects across all samples. Cell clustering was constructed using the " FindNeighbors” and " FindClusters” functions in the Seurat package, and visualized by the t-distributed stochastic neighbor embedding (t-SNE) method. Cell types’ annotation was conducted by referring to previously published marker genes for each cell type [21].

To differentiate malignant cells in ccRCC samples, we utilized normal kidney epithelial cells as a reference to assess the copy number variation (CNV) using the infercnv R package. Subsequently, we identified the differentially expressed genes (DEGs) between ccRCC cells and normal epithelial cells by employing the “FindMarkers” function and we defined the upregulated genes in tumor cells (log2FC > log2(1.5), FDR < 0.05) as malignant epithelial cell-related genes (MECRGs). KEGG (Kyoto Encyclopedia of Genes and Genomes) and GO (gene ontology) pathway analysis is a bioinformatics approach used to interpret large-scale gene expression data in the context of biological pathways. To gain further insights into the biological significance of these MECRGs, we conducted enrichment analyses for GO and KEGG pathways.

### Construction and validation of machine learning-based signature

To construct a reliable prognostic model with strong predictive accuracy, we utilized an extensive method that integrated 101 varied combinations of 10 distinct machine learning algorithms. The integrated algorithms consisted of the following: stepwise Cox, Lasso, Ridge, CoxBoost, random survival forest (RSF), elastic network (Enet), partial least squares regression for Cox (plsRcox), generalized boosted regression modeling (GBM), supervised principal components (SuperPC), and survival support vector machine (survival-SVM). Importantly, several of these algorithms, such as Lasso, stepwise Cox, RSF, and CoxBoost, were equipped with feature selection capabilities.

The procedure for generating the prognostic signature could be summarized as follows:

- Identifying the Prognostic MECRGs: Prognostic MECRGs were identified in the TCGA-KIRC cohort using univariate Cox regression.
- Developing prognostic model: Next, 101 combinations of algorithms were applied to the prognostic MECRGs for variable selection and model construction based on the tenfold cross-validation framework.
- Testing models’ performance in validation cohorts: All models were evaluated in E-MTAB-1980, E-MTAB-3267, CPTAC, GSE22541, and GSE29609 datasets.
- Choosing the best model: The Harrell’s concordance index (C-index) for each model across all datasets was calculated, and the model with the high average C-index and clinically translational significance was considered optimal. As a result, the optimally prognostic model was called malignant epithelial cell-related genes’ signature (MECRGS).

To evaluate MECRGS, we conducted a thorough literature search on PubMed to collect the published signatures predicting ccRCC outcomes. Next, we computed MECRGS scores for six cohorts utilizing the genes or RNA along with the coefficients supplied in the corresponding articles. Finally, we compared their performance in predicting the overall survival (OS) of ccRCC using the C-index.

### Evaluation of immune cell infiltration patterns

After applying the optimal model, patients were categorized into high- or low-score groups using the cut-off MECRGS score in all cohorts. we analyzed the infiltration of immune cells and immune-related signatures in each group using various algorithms. These included single-cell gene set enrichment analysis (ssGSEA) [22], MCPcounter [23], EPIC [24], XCELL [25], CIBERSORT [26], ESTIMATE [27], TIMER [28], and QUANTISEQ [29]. These algorithms allowed us to assess and quantify the presence of immune cells within the tumor microenvironment. We further compared the expression levels of marker genes associated with immune modulation, which include co-inhibitors, co-stimulators, major histocompatibility complex (MHC) genes, and chemokines (Table S1) [30].

### Gene set variation analysis (GSVA)

For bulk RNA cohorts, we conducted gene set variation analysis (GSVA) [22] using immune cell-related signatures obtained from prior studies (Table S2, S3) [31, 32]. As for the single-cell RNA datasets, we employed GSVA with hallmark gene sets from the MSigDB database (Table S4) to identify enriched pathways in various clusters of tumor cells. Afterward, the enrichment scores were computed for each pathway included in the gene sets.

### Tumor stemness and cell-cell communication analysis

The malignant cells were further clustered using a resolution parameter set to 0.3, leading to the identification for different subtypes of cancer cells exhibiting distinct gene expression patterns. The CytoTRACE R package [33], which is designed to quantify stemness with superior performance, was utilized to calculate the CytoTRACE scores for the malignant cells. The CytoTRACE score, ranging from 0 to 1, inversely correlates with the level of differentiation: a higher score signifies lower differentiation (greater stemness), while a lower score suggests higher differentiation.

To understand the intercellular communications between immunocytes, stromal cells, and malignant cells, we explored the potential signaling interactions mediated through ligand-receptor pairs by the ‘CellChat’ R package [34].

### Analysis of the immunotherapeutic effects between two MECRGS score groups

Patients who received nivolumab treatment were gathered from the immunotherapy cohort CheckMate025 [35] to assess the predictive capacity of our signature. Anti-PD-1/PD-L1 therapy scRNA cohorts [36] were downloaded to further explore the mechanisms underlying the response immunotherapy.

### Immunohistochemistry (IHC) and multiplex immunofluorescence (mIF)

For the Renji cohort, Immunohistochemistry (IHC) analysis was conducted using Tissue microarray (TMA) tissue samples with approved ethics [37]. The protocol involved the following steps: First, the TMA sections were incubated with primary antibodies against Anti-PLOD2 antibody (rabbit, 21214-1-AP, Proteintech); Anti-SAA1 antibody (rabbit, A1655, Abclonal); Anti-C1S antibody (rabbit, 14554-1-AP, ProteinTech); Anti-C1R antibody (rabbit, A25032, Abclonal). Next, a peroxidase affineur goat secondary antibody (111-035-003, JACKSON) was applied to the sections. After washing, the sections were stained with DAB (Sigma-Aldrich, D8001) and hematoxylin for signal visualization.

Next, the Renji TMA slides were subjected to multiplex immunofluorescence (mIF) staining. The primary antibodies used were: Anti-PLOD2 antibody (rabbit, 21214-1-AP, Proteintech); Anti-SAA1 antibody (rabbit, A1655, Abclonal); Anti-CA9 antibody (rabbit, 11071-1-AP, ProteinTech); The secondary antibody and tyramide signal amplification (TSA) (ZCTS002_20, ZCTS004_20) were used for incubation and signal amplification. The nuclei were counterstained with DAPI (D9542, Sigma). Subsequently, images were acquired using the PANNORAMIC MIDI II (3D HISTECH). Following this, quantification of the stained markers was conducted, enabling the analysis of marker expression in the ccRCC samples.

### Statistical analysis

Data analysis and graphical visualization were conducted using R (version 4.1.4). Continuous variables were analyzed using either the Wilcoxon rank-sum test or Student’s t-test. For categorical variables, the Chi-square test or Fisher’s exact test was used for comparison. Survival analysis was conducted using the survival package, including univariate and multivariate Cox regression analysis as well as Kaplan-Meier survival analysis. To evaluate the performance of the model, the timeROC package was utilized to construct the receiver operating characteristic (ROC) curve. *P* value less than 0.05 was generally considered statistically significant (*P < 0.05, **P < 0.01, ***P < 0.001, ns: not significant).

## Results

### Identification of the malignant epithelial cell-related genes using scRNA-seq data

**Fig. 1:**
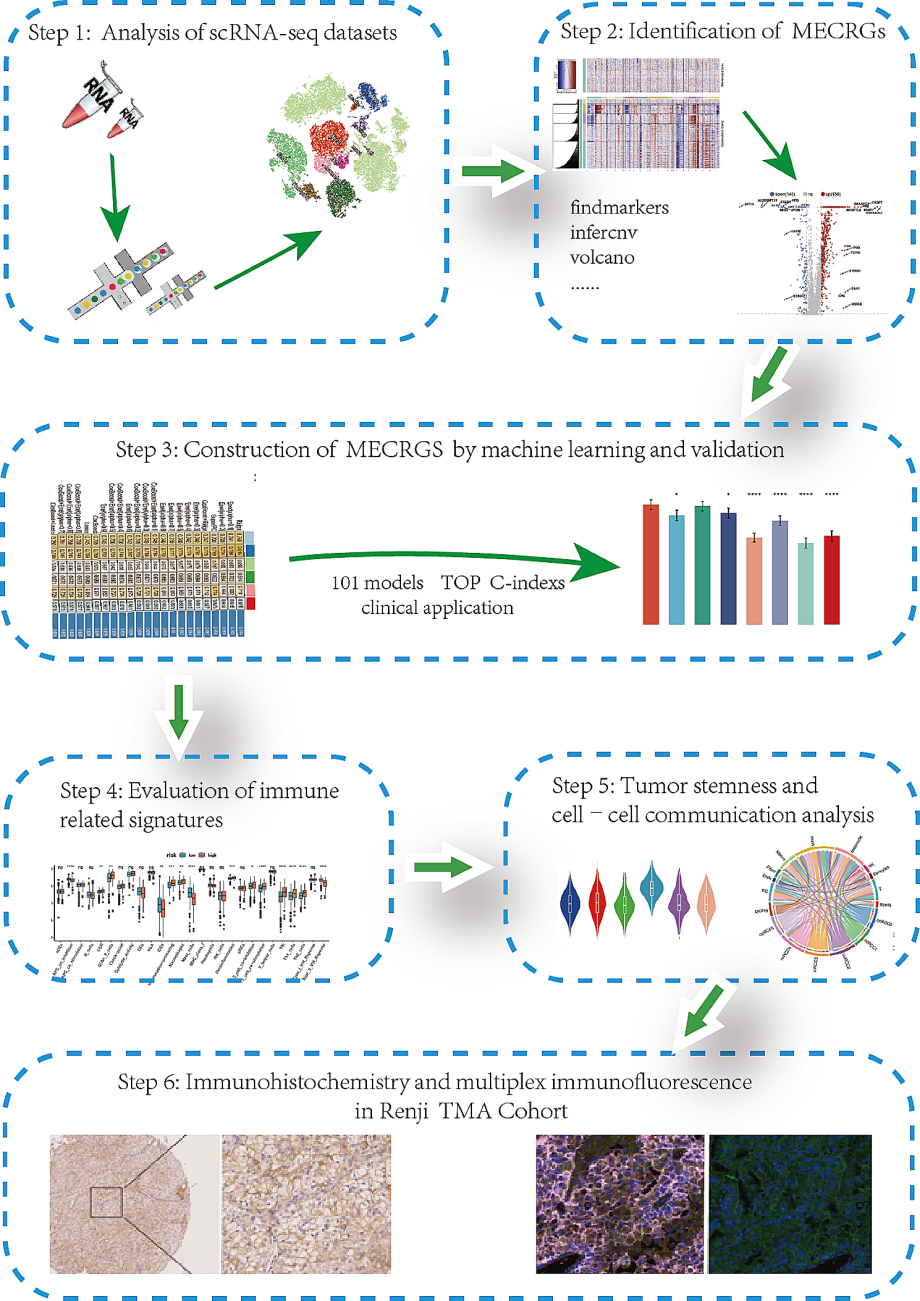
The flowchart of the overall study

**Fig. 2:**
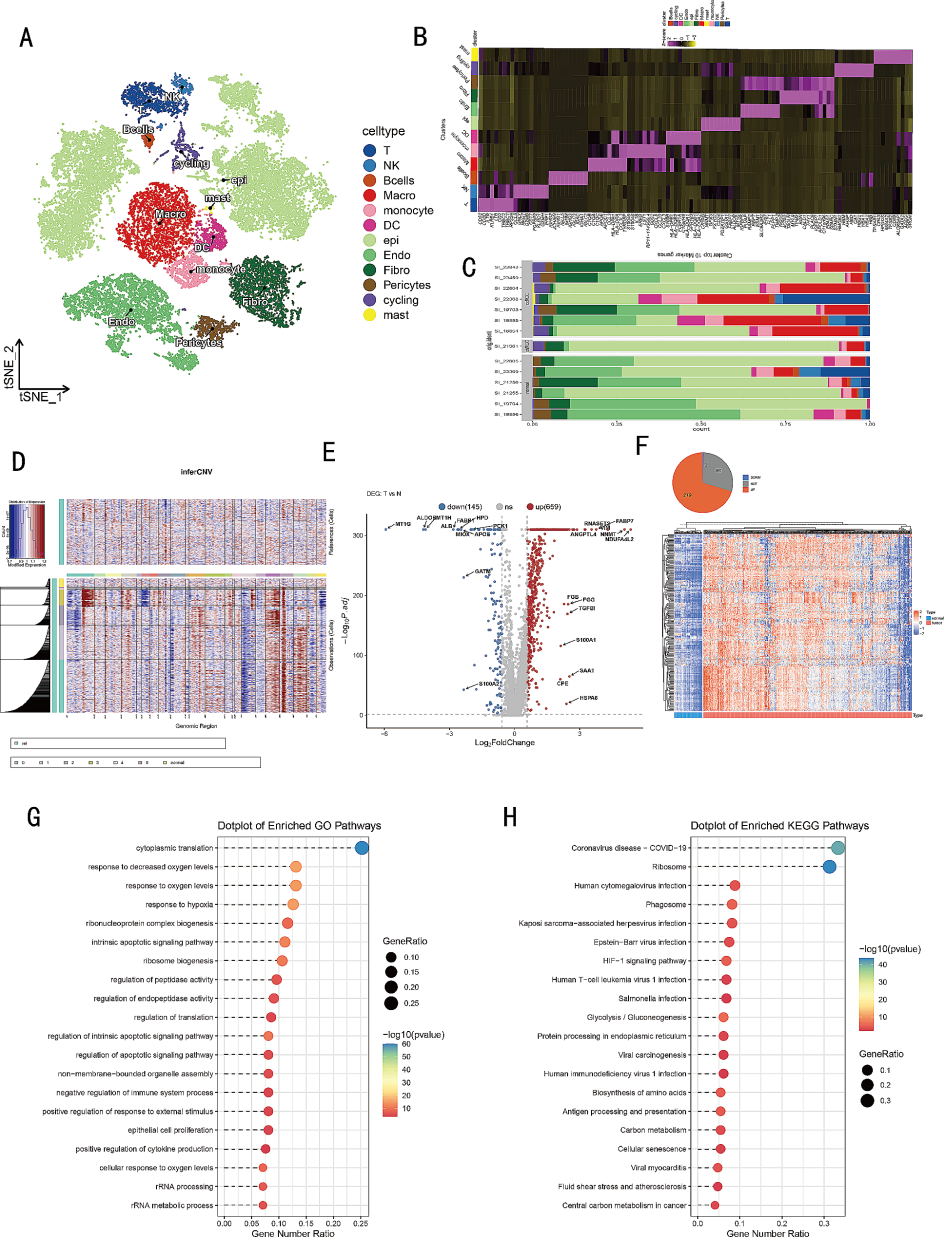
Identification the DEGs between cancer cells and normal epithelial cells. (**A**) *t*-SNE plot demonstrating the distribution of different cell types. (**B**) Heatmap showing the top genes highly expressed in different cell types. (**C**) Bar plot showing cell population distributions across tumor and normal samples. (**D**) CNV landscape revealing differences between malignant and normal cells. (**E**) Volcano plot of DEGs between malignant and non-malignant cells. (**F**) Pie plot of DEGs and heatmap of 219 MECRGs in TCGA- KIRC dataset. (**G**, **H**) GO and KEGG enrichment of the 219 common MECRGs.

The flowchart of our study was shown in Fig. 1. To explore the genes highly expressed in malignant epithelial cells of ccRCC compared to normal kidneys, we conducted single-cell RNA sequencing (scRNA-seq) analysis on 8 RCC tumors and 6 benign human kidney tissues obtained from previously published research (GSE159115) [21]. Out of the eight patients, seven were diagnosed with ccRCC, while one had chromophobe renal cell carcinoma (chRCC), which was excluded from further analysis. After quality control mentioned in methods, we utilized the harmony R package to remove batch effects, and subsequently, we performed t-Distributed Stochastic Neighbor Embedding (t-SNE) dimensionality reduction for visualizing the scRNA-seq data with a clustering criterion of 0.8, as depicted in Fig. S1A. Then we assigned the 25 subpopulations to 11 distinct cell types using marker genes as references Fig. S1B. These cell types include epithelial cells, fibroblast/pericytes, endothelial cells, macrophages, monocytes, dendritic cells, mast cells, cycling cells, T cells, NK cells, and B cells (Fig. 2A). The heatmap in Fig. 2B demonstrated the top marker genes for each cell population. The percentage of different cell types varied greatly among tumor and normal tissues, and epithelial cells constituted a significant proportion of the total cell population (Fig. 2C). Utilizing the InferCNV function, we distinguished malignant cells from normal cells, classifying all epithelial cells derived from the tumor as malignant cells for they exhibited high levels of copy number variation (CNV) (Fig. 2D). Our findings aligned with previous studies reporting frequent aberrations in ccRCC, such as the deletion of chr 3p and 14, and the amplification of chr 5q [38]. Consistent conclusions were obtained from two additional datasets: GSE210042 and GSE156632 (Fig. S1C-F). After performing differential analysis to compare malignant and non-malignant epithelial cells in ccRCC, we were able to identify 659 genes that were upregulated in malignant cells (Fig. 2E). Intersecting the upregulated genes with those from GSE210042 and GSE156632 (Fig. S1G, H), we obtained 311 consistently upregulated genes in malignant epithelial cells (Fig. S1I). Upon utilizing TCGA KIRC bulk RNA-seq data for validation, we found that 219 of these genes remained highly expressed in tumors at the overall level (Fig. 2F). Consequently, we designated these genes as malignant epithelial cell-related genes (MECRGs). The GO analysis indicated these MECRGs were significantly enriched in pathways associated with responding to decreased oxygen levels, response to hypoxia, and negative regulation of the immune system process (Fig. 2G, Table S5). Additionally, KEGG pathway analysis revealed significant enrichment in pathways related to HlF-1 signaling pathway, antigen processing and presentation, as well as glycolysis/gluconeogenesis (Fig. 2H, Table S6). These results are consistent with previous studies, indicating a close association between hypoxia and the development of ccRCC tumors [39, 40]. This consistency underscores the accuracy of the MECRGs we identified and highlights their relevance in the context of cancer biology.

### Integrated development of a MECRGS and evaluation

**Fig. 3:**
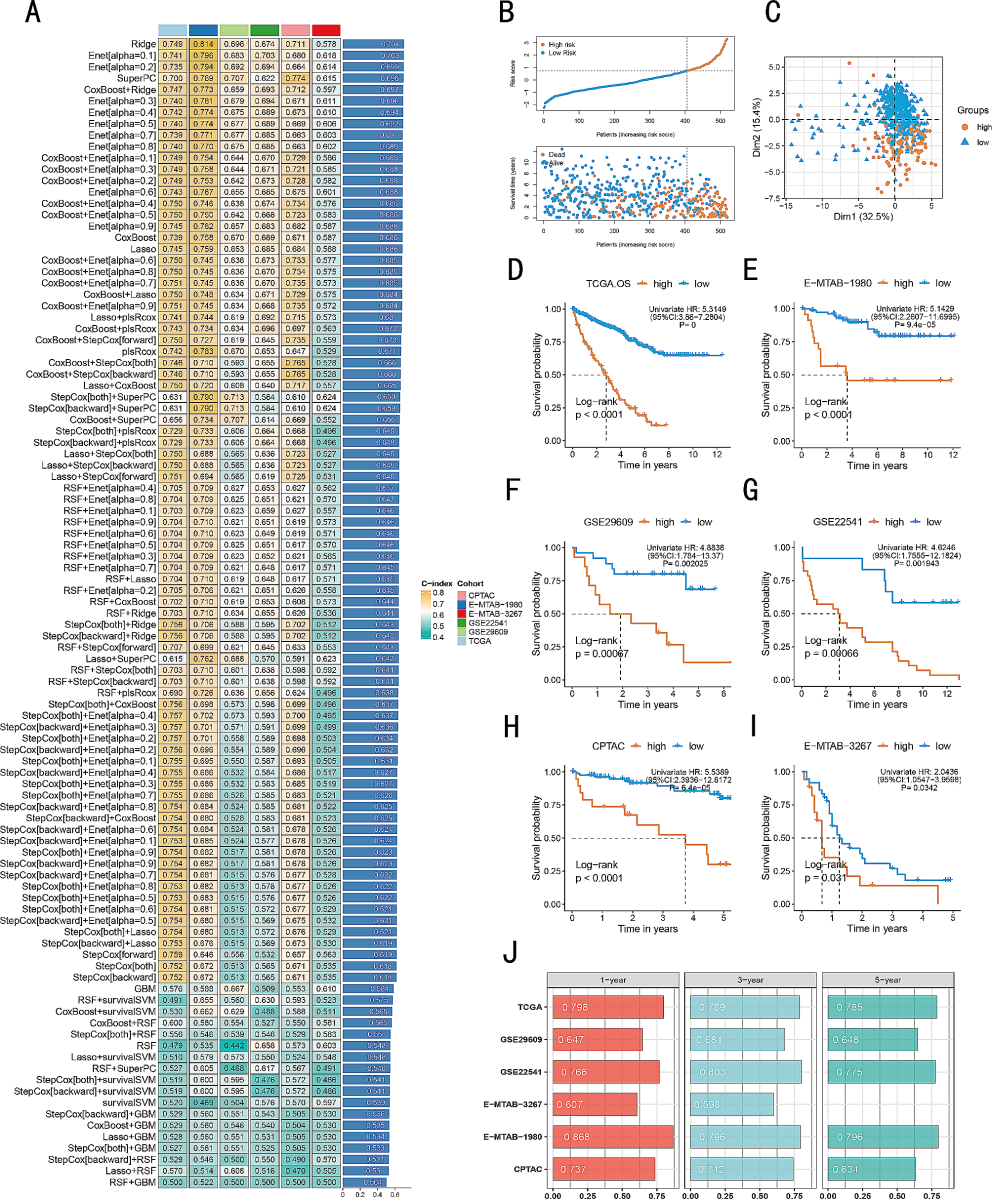
Development and validation of the machine learning-based prognostic signature, MECRGS. (**A**) C-index of 101 prediction models using 10 machine learning algorithms across 6 cohorts. (**B**) Patient distribution based on MECRGS scores and survival status. (**C**) PCA plots showing the distribution of low- and high- MECRGS groups. (**D**-**I**) Kaplan– Meier survival analysis of low- and high- MECRGS groups in different cohort. (**J**) Time-dependent ROC analysis for predicting OS at 1, 3, and 5 years

Initially, we conducted a univariate Cox analysis on 219 MERGs, and identified 71 prognostic-related genes (Table S7). These 71 genes further underwent a machine learning-based integrative method to determine the optimal model with the highest accuracy and stability. Out of the 101 machine learning models, the top five ranked models demonstrated high average concordance index (C-index) values (Fig. 3A). The Ridge, Enet, and SuperPC models incorporated all 71 genes, whereas the “CoxBoost + Ridge” model only utilized 27 genes. After thorough evaluation, we ultimately chose the “CoxBoost + Ridge” model as our final selection due to its exceptional performance and simplicity. Finally, we developed a malignant epithelial cell-related signature (MECRGS) based on the combined CoxBoost and Ridge models, which exhibited high accuracy across multiple datasets.

**Fig. 4:**
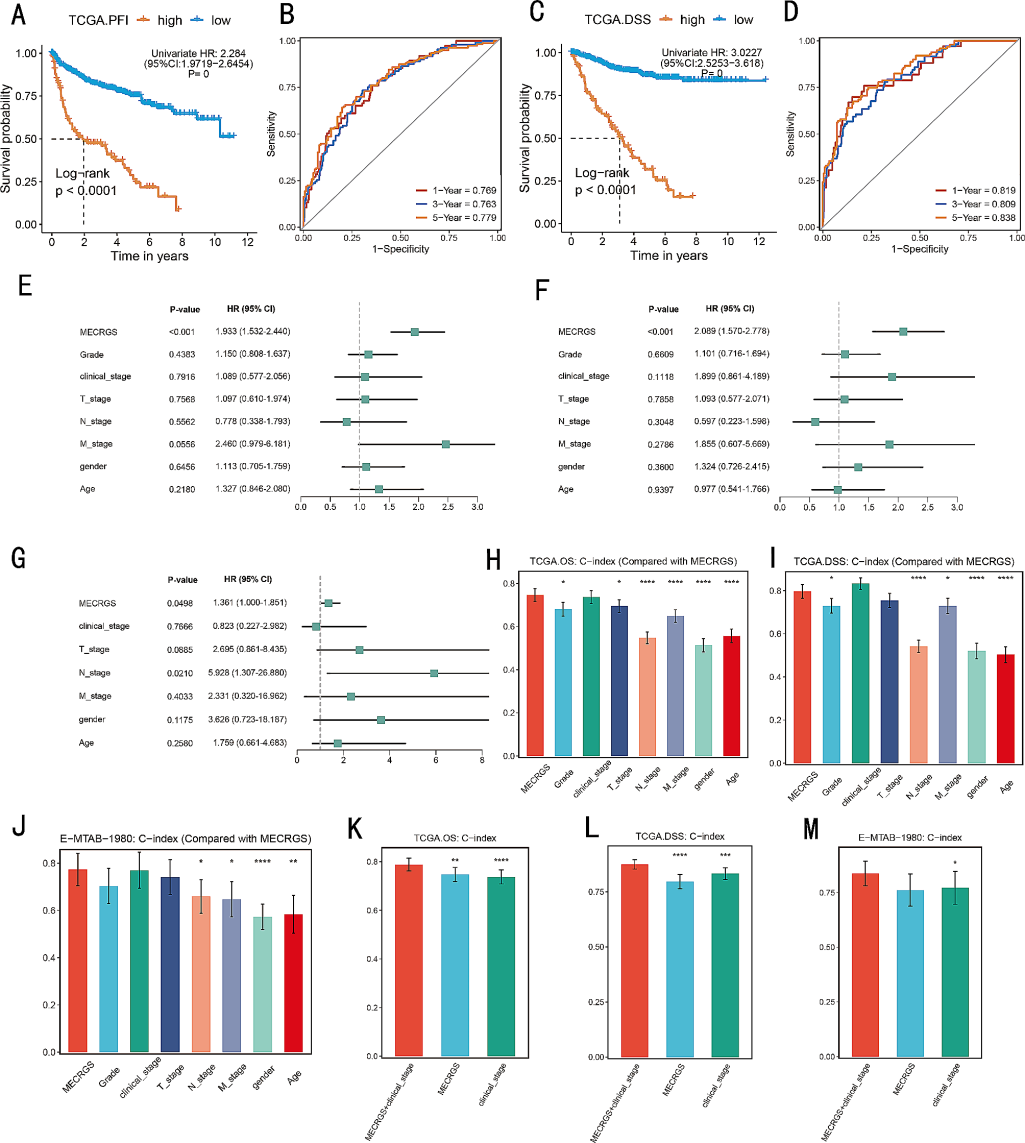
Survival analysis and predictive performance evaluation of MECRGS. (**A**-**D**) Kaplan–Meier survival analysis and time-dependent ROC analysis for PFI (**A**, **B**) and DSS (**C**, **D**). (**E**-**G**) Multivariable Cox regression analysis of OS and PFI in TCGA- KIRC dataset (**E**, **F**) and E-MTAB-1980 cohort (**G**). (**H**-**M**) The performance of MECRGS compared with other clinical related variables in predicting prognosis in TCGA- KIRC and E-MTAB-1980 cohorts

According to the optimal cut-off score calculated by survminer package, ccRCC patients were divided into high-MECRGS score group and low-MECRGS score group (Fig. 3B). The PCA algorithm results indicated that patients in the TCGA-KIRC cohort could be accurately classified using the MECRGS model genes, as shown in Fig. 3C. Across all datasets, patients with high MECRGS score had significantly shorter overall survival (Fig. 3D-I). Subsequently, we conducted a time-dependent ROC analysis to evaluate the discriminative ability of MECRGS in terms of survival (Fig. 3J). The TCGA-KIRC training cohort showed the areas under the ROC curve (AUC) values of 0.798, 0.789, and 0.785 for the overall survival (OS) at 1-year, 3-year, and 5-year time points, respectively. Additionally, remarkable performance was observed in the five testing cohorts: E-MTAB-1980 had values of 0.868, 0.796, and 0.796 for 1-year, 3-year, and 5-year OS, respectively; CPTAC had values of 0.737, 0.742, and 0.634; GSE22541 had values of 0.766, 0.803, and 0.775; GSE29609 had values of 0.647, 0.681, and 0.648; and E-MTAB-3267 had values of 0.607 and 0.598 for 1 and 3 years, respectively. Furthermore, we found that patients in the low MECRGS group had a significantly longer progression-free interval (PFI) and disease-specific survival (DSS) compared to those in the high MECRGS group (*p* < 0.001, log-rank test; Fig. 4A, C). Additionally, the AUC values supported the accuracy of the MECRGS model in predicting survival outcomes (Fig. 4B, D). Overall, these results further highlighted the remarkable performance of MECRGS across multiple independent cohorts.

### Relation of MECRGS and clinical characteristics

In clinical practice, clinical traits were often used to assess the prognosis of ccRCC patients. In our study, we aimed to evaluate the correlation between the MECRGS and various clinical traits. In the training dataset, we identified notable variations in the distribution of clinical stage, pathological grade, T stage, N stage, and M stage between the high- and low-MECRGS subgroups (*p* < 0.0001, Fig. S2A-E). It was observed that patients with high clinical or pathological stages tended to have high MECRGS scores, indicating potential association between the MECRGS and disease progression. Interestingly, we also found that the MECRGS could predict the presence of metastases in ccRCC patients with an AUC of 0.748 (Fig. S2F). These results were further confirmed in the validation cohorts (Fig. S2G-T), providing additional support for the association between the MECRGS and clinical traits related to poor prognosis and metastasis development.

Furthermore, multivariate Cox analysis demonstrated that MECRGS is an independent prognostic factor for OS, DSS and PFI in the training cohort after adjusting for clinical factors (Fig. 4E, F, Fig. S3A). This finding was validated in other datasets as well (Fig. 4G Fig. S3B, C), indicating the robustness of MECRGS as a prognostic marker for ccRCC. To further evaluate the superiority of MECRGS compared to other clinical-pathological features, we conducted a comprehensive comparison in both training and validation cohorts. The results demonstrated that MECRGS and clinical stage exhibited better predictive ability compared to each individual feature (Fig. 4H-J; Fig. S3D-F). Moreover, when we combined MECRGS with clinical stage, the integrated model showed a significantly higher C-index than using MECRGS or clinical stage alone (Fig. 4K-M; Fig. S3G, H). Therefore, the combination of MECRGS and clinical stage may further enhance the performance of our predictive model.

### Comparison of our prognostic signatures with 92 previously published signatures in ccRCC

In recent years, with the rapid development of bioinformatics and next-generation sequencing, an increasing number of prognostic models have been established in ccRCC. In our study, we collected 92 prognostic features involving various biological characteristics such as necroptosis, cuproptosis, oxidative stress, autophagy, fatty acid metabolism, and glutamine metabolism (Table S8). Across multiple datasets including TCGA, E-MTAB-1980, CPTAC, GSE29609, GSE22541, and Meta cohorts (comprising all patients from the collected datasets), our MECRGS demonstrated significantly better accuracy compared to other models ranking among the top five in all six cohorts (Fig. S4A), highlighting the robustness of MECRGS. It is worth noting that various prognostic features showed higher C-index in the TCGA-KIRC training cohort but performed poorly in other cohorts, potentially due to overfitting and compromised generalizability.

### MECRGS sculptured an inflamed but immunosuppressive TME of ccRCC

**Fig. 5:**
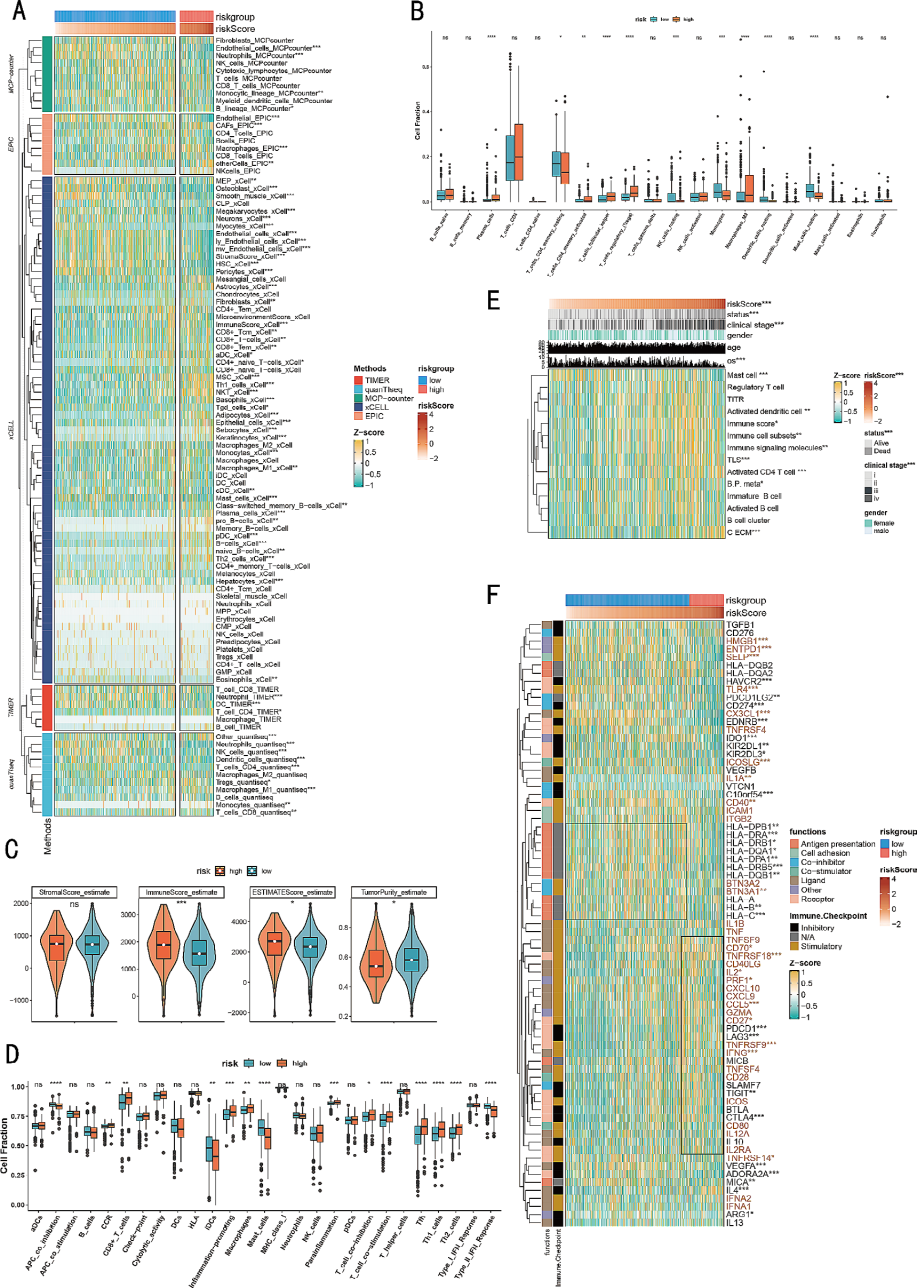
Alterations of immune infiltration and immunomodulators between two subgroups. (**A**, **B**) The differences in infiltration profiles of multiple immune cells between two clusters evaluated by several algorithms including EPIC, MCP-counter, QUANTISEQ, TIMER, xCell, and CIBERSORT, and then normalized and scaled into Z-score. (**C**) The ESTIMATE score, immune score, stromal score and the tumor purity calculated by ESTIMATE R package. (**D**, **E**) The comparison of several immune signatures in two subgroups. (**F**) The expression profiles of immune regulators for the Wilcoxon rank- sum test (***p < 0.001, ** *p* < 0.01, * *p* < 0.05)

We employed the IOBR R package to comprehensively analyze the infiltration of immunocytes in high and low MECRGS subgroups in TCGA-KIRC cohorts. Our findings revealed that immune cell infiltration levels, including activated CD4 + memory T cells, follicular helper T cells, CD8 + T cells (Tem cells, Tcm cells), NKT cells, plasma cells, and macrophages, were significantly higher in high-MECRGS patients, indicating an immune activation state (Fig. 5A, B). We also found that the immune scores obtained by the ESTIMATE package were higher in the high-MECRGS group. In contrast, the low-MECRGS group showed high enrichment of endothelial cells, smooth muscle cells, and other stromal cells. Nonetheless, there was no substantial distinction in stromal scores observed between the high and low MECRGS groups (Fig. 5C).

Additionally, we compared the infiltration abundance of the immune microenvironment with previously published signatures [41, 42]. We discovered that the high-MECRGS group not only showed elevated levels of immunocytes and immune signatures associated with chemokine receptors (CCR), T cell co-stimulation, and inflammation-promoting (Fig. 5D), but also exhibited significant activation of regulatory T cells (Tregs), cancer-associated extracellular matrix (C-ECM) and tumor-infiltrating regulatory T cells (TITR) signatures (Fig. 5E), indicating an exhausted and suppressive immune status.

Then, we compared the expression profiles of immune regulators and found that the high-MECRGS group was enriched with exhausted CD8 + T cell markers [43] such as PDCD1, LAG3, TIGIT, and CTLA4 (Fig. 5F). They also functioned as immune checkpoints. This finding indicates that the high MECRGS group exhibited elevated expression of immune checkpoint molecules compared to the low group, potentially to evade immune destruction following immune activation. On the other hand, the low-MECRGS group showed high expression levels of antigen presentation markers in the major histocompatibility complex (MHC) class II pathway, such as HLA-DRA, HLA-DRB1, HLA-DQA1, HLA-DPA1, HLA-DRB5, and HLA-DQB5. These markers are important for presenting antigens to CD4 + T cells and play crucial roles in anti-tumor immunity [44].

### Investigating hub genes of MECRGS at the single-cell level

**Fig. 6:**
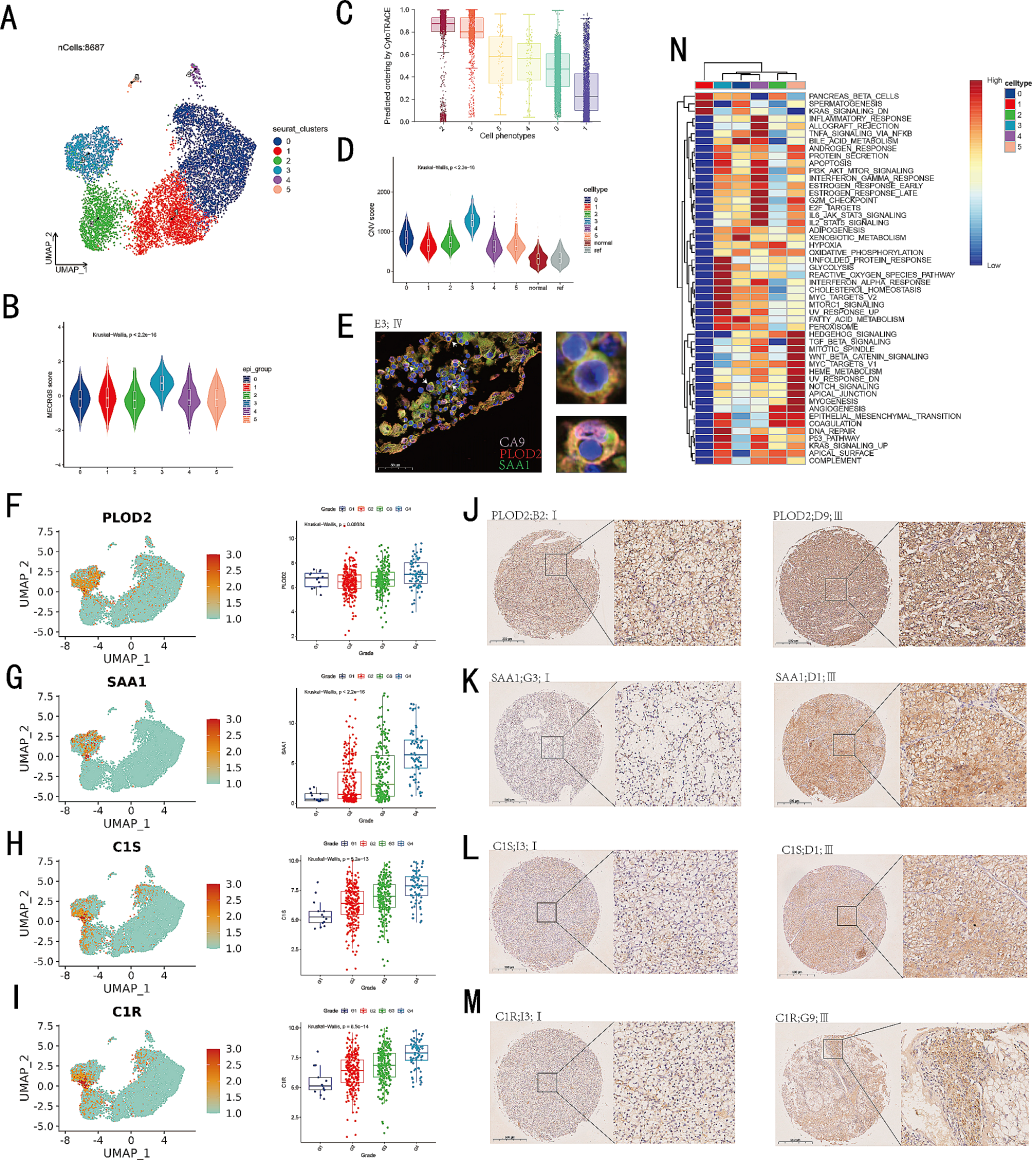
Intertumoral heterogeneity of tumor epithelial cells in ccRCC. (**A**) UMAPs of all malignant epithelial single-cell transcriptomes color-coded by cell types. (**B**) Violin plot of the MECRGS scores in different cancer cell clusters. (**C**) Box plot showing CytoTRACE scores among cancer cells. (**D**) CNV scores revealing the differences between different malignant cell clusters. (**E**) Multicolor immunofluorescence staining of CA9, PLOD2 and SAA1 in the Renji TMA cohort. Scale bar: 50 μm. Color: CA9 (pink), PLOD2 (red), SAA1 (green), and cell nucleus (blue). Arrow: PLOD2 + SAA1 + CA9 + cells. (**F**-**I**) Feature plots of PLOD2, SAA1, C1S, C1R (left); The PLOD2, SAA1, C1S, C1R expression levels were positively related with tumor stages (right). (**J**-**M**) IHC images of C1S, C1R, PLOD2, and SAA1 between low and high pathology grades in the Renji TMA cohort. Scale bar: 100 μm. Left: patients with low pathology grades; right: patients with high pathology grades. (**N**) Gene ontology enrichment of all components of cancer cells

To explore the function of MECRGS within the tumor microenvironment (TME) at the single-cell transcriptome level, we conducted an analysis of the expression profiles of hub genes across different cell types. The findings indicated that *C1S*, *C1R*, and *PLOD2* were predominantly expressed in malignant epithelial cells (Fig. S5A). They were also identified as significant risk factors in bulk RNA datasets (Fig. S5B). Then, we clustered tumor cells using the FindClusters function with a resolution parameter of 0.3 in GSE159115, and we identified a tumor subtype, PLOD2 + SAA1 + ccRCC cells, which showed higher MECRGS scores, CNV scores, and stemness scores (Fig. 6A-D, Fig. S6A), suggesting a malignant status. Furthermore, we observed the presence of this specific cluster of tumor cells in four additional scRNA sequencing datasets (Fig. S6B-E) and the Renji TMA cohort (Fig. 6E), characterized by elevated expression levels of *PLOD2*, *SAA1*, *C1R*, and *C1S*. Importantly, the expression of these genes increased with the advancement of TNM stage in ccRCC patients (Fig. 6F-M), suggesting that they could serve as a valuable prognostic indicator in ccRCC. These findings also indicated that the upregulation of these genes may be associated with the progression and aggressiveness of ccRCC. Moreover, the GSVE analysis of Hallmark pathways showed that this subtype of tumor cells demonstrated heightened activity in pathways associated with epithelial-mesenchymal transition, glycolysis, reactive oxygen species pathway, interferon alpha response, and complement (Fig. 6N). The GO and KEGG pathway enrichment analysis revealed this cluster was enriched in the regulation of angiogenesis, as well as in the complement and coagulation cascades (Fig. S6F, G).

### PLOD2 + SAA1 + tumor cells’ communication with other cell types

**Fig. 7:**
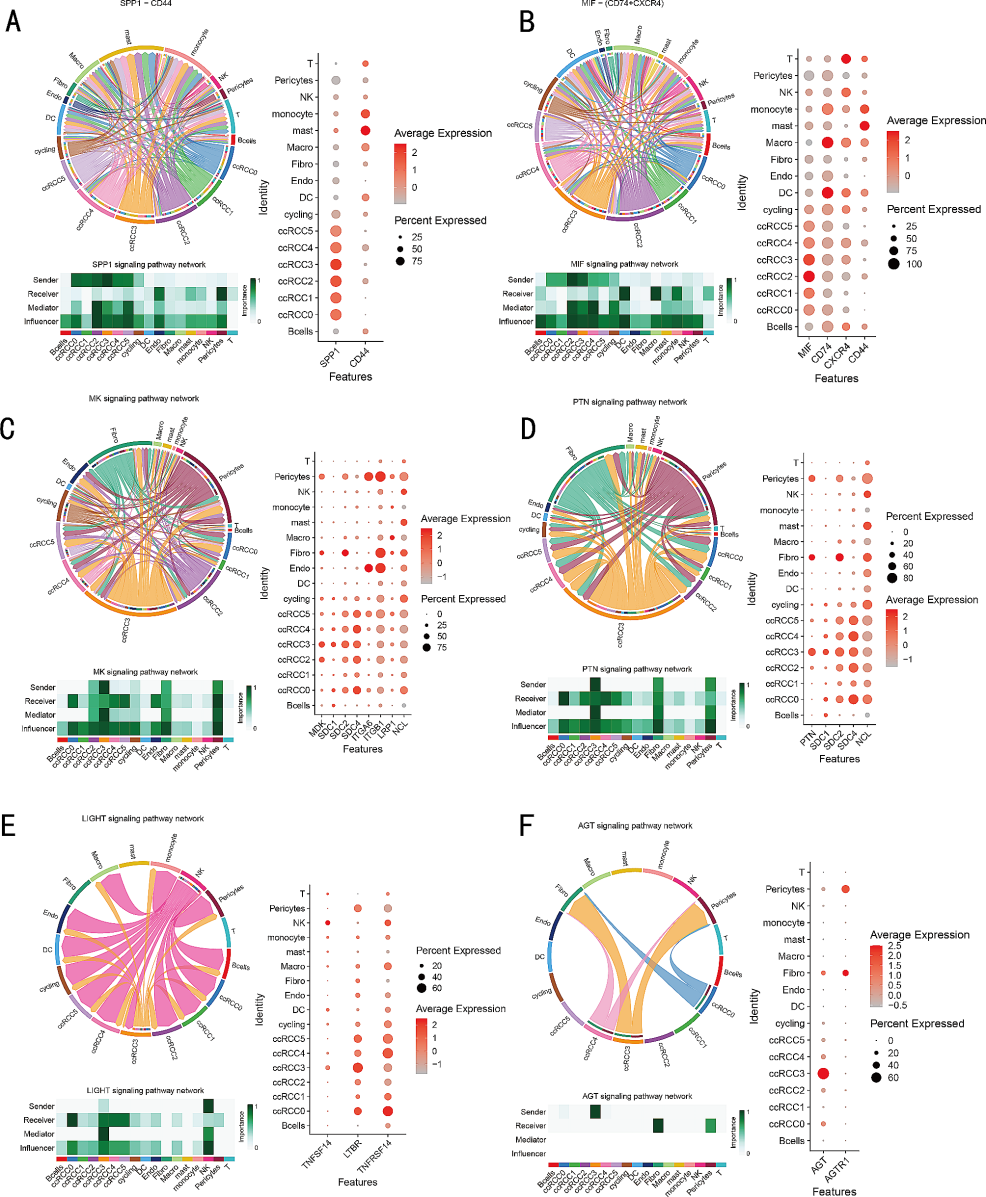
Cell–cell communication between cancer cells and other cell types. (**A**-**F**) Chord diagrams (left) of SPP1, MIF, MK, PTN, LIGHT and AGT signaling pathways showing complex interactions between cell populations; Heatmaps (bottom) depicting the roles of different cell types playing in the pathway network; Dotplot (right) depicting the differential expression of signaling L-R pair genes in different cell types

**Fig. 8:**
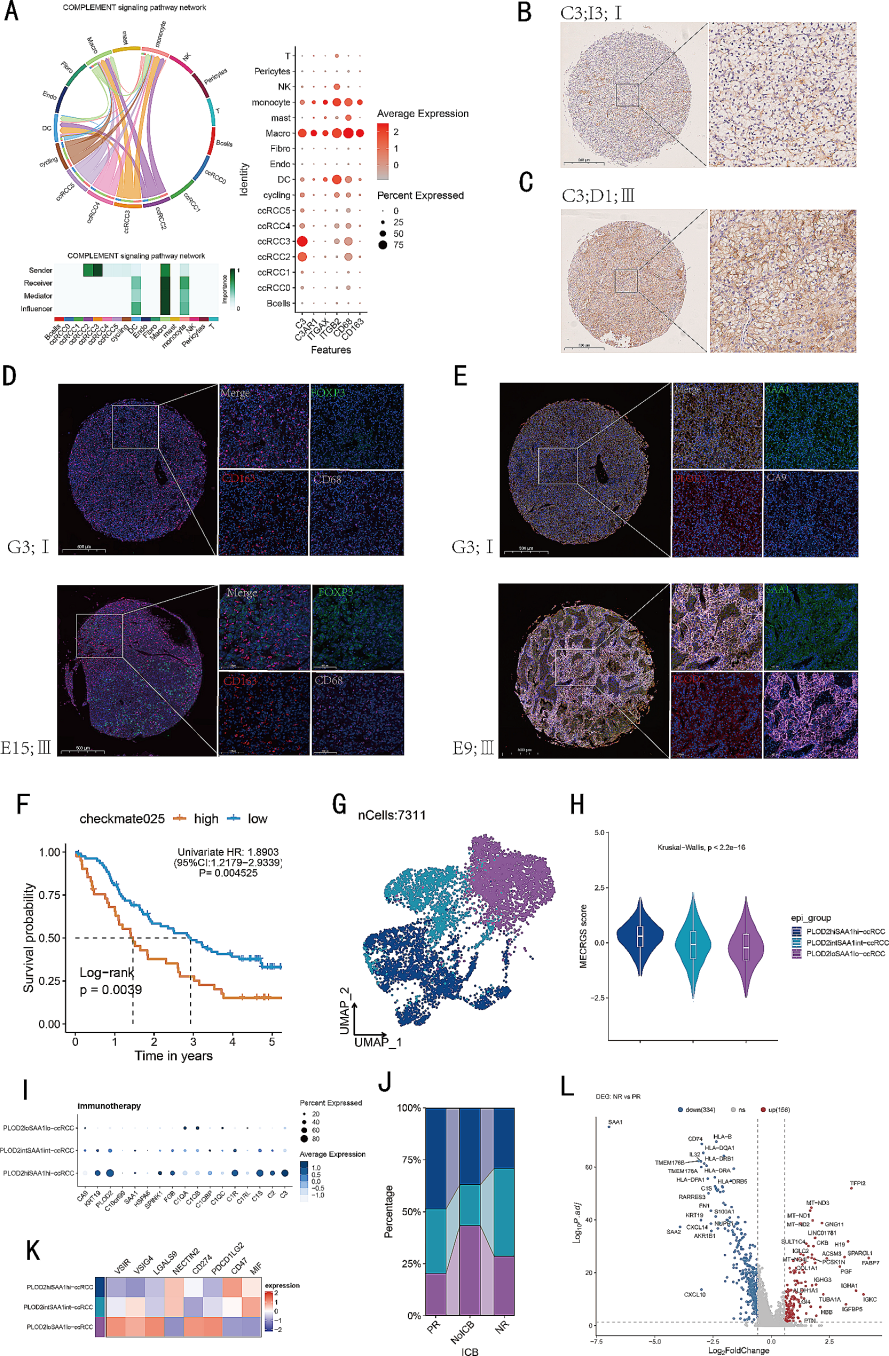
Exploration of the C3-C3AR1 pairs and the significance of MECRGS in immunotherapy cohort. (**A**) The communications between PLOD2 + SAA1 + cancer cells and other cell types through complement signaling pathway. (**B**, **C**) IHC images of C3 between low and high pathology grades in the Renji TMA cohort. Scale bar: 100 μm. (**D**) Multicolor IF images of CD68, CD163 and FLOX3 between low and high pathology grades in the Renji TMA cohort. Scale bar: 100 μm. CD68 + cell (pink), CD163 + cell (red), FLOX3 + cell (green), and cell nucleus (blue). (**E**) Multicolor IF images of CA9, PLOD2 and SAA1 between low and high pathology grades in the Renji TMA cohort. Scale bar: 100 μm. CA9 + cell (pink), PLOD2 + cell (red), SAA1 + cell (green), and cell nucleus (blue). (**F**) Kaplan– Meier survival analysis of low- and high- MECRGS groups in CheckMate025 cohort. (**G**) Umap plot demonstrating the distribution of three tumor cell types. (**H**) Violin plot of the MECRGS scores in different cancer cell clusters. (**I**) Dot plots illustrating the distribution of marker genes across three tumor cell clusters. (**J**) Heatmap plot showing the expression of immune checkpoints across three tumor cell clusters. (**K**) Bar plot showing cell population distributions across PR, NR and NoICB samples. (**L**) Volcano plot showing the DEGs between NR and PR patients after immunotherapy

Next, we investigated PLOD2 + SAA1 + tumor cells’ interactions with other types of cells in the TME. Cellular responses are activated by ligand-receptor interactions, leading to the activation of specific signaling pathways. Based on our findings, we observed that PLOD2 + SAA1 + tumor cells were able to communicate with TME cells and act as strong senders and influencers in signaling pathways such as SPP1, MIF, PTN, and MK. They also acted as receivers in the LIGHT signaling pathway, while acting as senders in the AGT and COMPLEMENT signaling pathway (Figs. 7A-F and 8A). The LIGHT signaling pathway involves the interaction between the tumor necrosis factor (TNF) family member called LIGHT (lymphotoxin-related inducible ligand) and its receptors, such as LTβR (lymphotoxin beta receptor). This pathway plays a role in inflammation, immune responses, and lymphoid organ development [45]. Our research indicates that PLOD2 + SAA1 + tumor cells might be capable of communicating with tumor-associated macrophages (TAMs) through specific ligand-receptor pairs, such as MIF-(CD74 + CXCR4), MDK-LRP1 and C3-C3AR1. In our immunohistochemistry (IHC) and multiplex immunofluorescence (mIF) analysis of the Renji TMA cohort, we observed that patients with advanced TNM stage had a higher infiltration of PLOD2 + SAA1 + tumor cells and elevated expression levels of *C3* (Complement component 3), which positively correlated with increased infiltration of CD163 + M2 TAMs and FOXP3 + regulatory T cells (Tregs) (Fig. 8B-E). FOXP3 + Tregs play a crucial role in maintaining immune tolerance and preventing autoimmunity [46]. These results suggest that PLOD2 + SAA1 + tumor cells may play a significant role in modulating the TME and influencing key signaling pathways involved in cancer progression.

### The role of MECRGS in immunotherapy

Based on the survival analysis in the E-MTAB-3267 clinical trial cohort of 53 ccRCC patients who received sunitinib treatment, the high-MECRGS group showed decreased survival time (Fig. 3I). In the CheckMate025 cohort (patients treated with Nivolumab), it was also observed that high-MECRGS group had a poorer prognosis (Fig. 8F, *p* < 0.01). To further assess the relevance of the MECRGS to immunotherapy, we investigated it in an immunotherapy scRNA dataset [36]. We observed that the percentage of PLOD2 + SAA1 + tumor cells with high MECRGS scores (Fig. 8G-J), as well as the expression of immune checkpoints such as *NECTIN2* and *CD47*, increased in patients who exhibited a partial response (PR) to immunotherapy (Fig. 8K). Following therapy, the PR patients displayed elevated expression levels of *RARRES3* and several MHC modules, which are crucial for immune responses [47]. However, we also noted an increase in the expression of certain tumor related genes, including *SAA1*, *KRT19* and *NUPR1* (Fig. 8L). Overall, these insights provide valuable information regarding the molecular mechanisms underlying the response to immunotherapy.

## Discussion

ccRCC is the most aggressive form of renal tumor, characterized by its malignant progression and high recurrence rate. With the advancement of sequencing technology, multiple predictive signatures have been developed for the precision therapy of ccRCC. However, their clinical utility was found to be limited [48]. This limitation underscores the necessity of developing a consensus signature to stratify patients across numerous cohorts. ccRCC exhibits high levels of heterogeneity, and its development is driven by complex epigenetic mechanisms and molecular pathways that vary between individuals [49]. The majority of RCC cases originate from the renal tubular epithelial cells, making it crucial to investigate the different gene expressions between the tumor cells and normal epithelial cells.

In this study, we identified 219 consistently expressed MECRGs by using scRNA-seq and bulk RNA-seq. Additionally, we selected 10 commonly used machine learning algorithms and utilized them to construct a total of 101 models. After careful evaluation, we determined that the combination of CoxBoost and Ridge regression——MECRGS, yielded the best performance in predicting patients’ prognosis, thanks to its simplicity and enhanced generalizability. Specifically, the model demonstrated high AUC values for the TCGA-KIRC, GSE22541, and E-MTAB-1980 cohorts, indicating consistent and robust performance across these datasets. However, the AUC values were not as high for the E-MTAB-3267 and GSE29609 cohorts, potentially due to the smaller sample sizes and additional treatments received by the patients in these cohorts, which may have contributed to variations in gene expression patterns, ultimately affecting the predictive ability of our model in these cohorts. Notably, the MECRGS model showed superior performance compared to 92 previously published signatures across multiple datasets. The MECRGS stood out as the only independent and prognostic indicator in all cohorts and surpassed many clinical indicators. In addition, we found the combination AJCC stage and our model may further enhance the predictive ability. Using the cut-off MECRGS score, patients were categorized into high or low- MECRGS subgroups. Patients with high MECRGS scores exhibited an immunogenically “cold” TME phenotype, characterized by an increased presence of immunosuppressive cells, and elevated levels of cytokines and chemokines with immunosuppressive effects. Additionally, these patients showed upregulation of immune exhaustion markers such as *LAG3*, *PDCD1*, *TIGIT*, *CTL4*, and C-ECM, TITR signatures, indicating a state of immune dysfunction.

Subsequent investigation of the hub genes of the MECRGS model at the single-cell RNA level revealed that *PLOD2*, *C1S*, and *C1R* were primarily expressed in tumor cells. *C1S* and *C1R* are genes that encode for a serine protease, which is a component of the C1 complex of the complement system. C1R cleaves and activates C1S, leading to the subsequent activation of downstream complement components [50]. Recent studies found that the complement system had the potential to contribute to immunosuppression and bolster the growth and invasiveness of tumors [51]. PLOD2 (procollagen-lysine,2-oxoglutarate 5-dioxygenase 2), also known as lysyl hydroxylase 2, is an enzyme involved in the post-translational modification of collagen. Collagen is a major component of the extracellular matrix and provides structural support to tissues. Additionally, it has been observed that the organization of collagen can influence the migration and invasion of cancer cells, essentially serving as a pathway or route for their movement [52]. Further clustering analysis of the tumor cells identified a subcluster characterized by high expression levels of *PLOD2*, *SAA1*, *C1S*, and *C1R*. Previous studies have indeed demonstrated that *PLOD2* expression can be induced by hypoxia, leading to the activation of the EGFR/AKT signaling pathway and subsequent promotion of proliferation and migration of ccRCC cells [53]. SAA1, also known as Serum Amyloid A1, is a protein that is produced primarily by the liver in response to inflammation. Elevated levels of SAA1 have been observed in certain types of cancers and are considered a potential biomarker for cancer progression and prognosis. Studies have found increased levels of SAA1 in cancers such as lung cancer [54], ovarian cancer [55], and breast cancer [56]. Additionally, the upregulation of *PLOD2* and *SAA1* has been shown to promote ccRCC development [53, 57]. Among the bulk RNA datasets, we observed a robust correlation between the upregulation of *PLOD2* and *SAA1* and poor prognosis in ccRCC patients. Furthermore, we confirmed this finding through validation in the Renji TMA cohort. Moreover, our investigation of single-cell RNA datasets revealed that tumor cells expressing high levels of *PLOD2* and *SAA1* exhibited enhanced malignancy, as indicated by elevated MECRGS scores, stemness, and copy number variations (CNVs). These particular cancer cells also activated complement pathways and secreted complements such as C1S, C1R, and C3, which facilitated the recruitment of tumor-associated macrophages (TAMs) and T cells [58]. Through mIF and IHC techniques applied to the Renji TMA cohort, we were able to identify the presence of PLOD2 + SAA1 + ccRCC cells and we found this tumor subclusters were related with high infiltration of CD163 + macrophages and Treg cells, indicating the establishment of an immunosuppressed TME. M2 macrophages, also referred to as alternatively activated macrophages, are a subset of immune cells known for their anti-inflammatory and immunoregulatory functions. CD163 is a membrane receptor predominantly expressed on the surface of M2 macrophages. CD163 + M2 TAMs always exhibit characteristics and functions associated with promoting tumor progression and are capable of secreting various cytokines that facilitate this process [59].

**Fig. 9:**
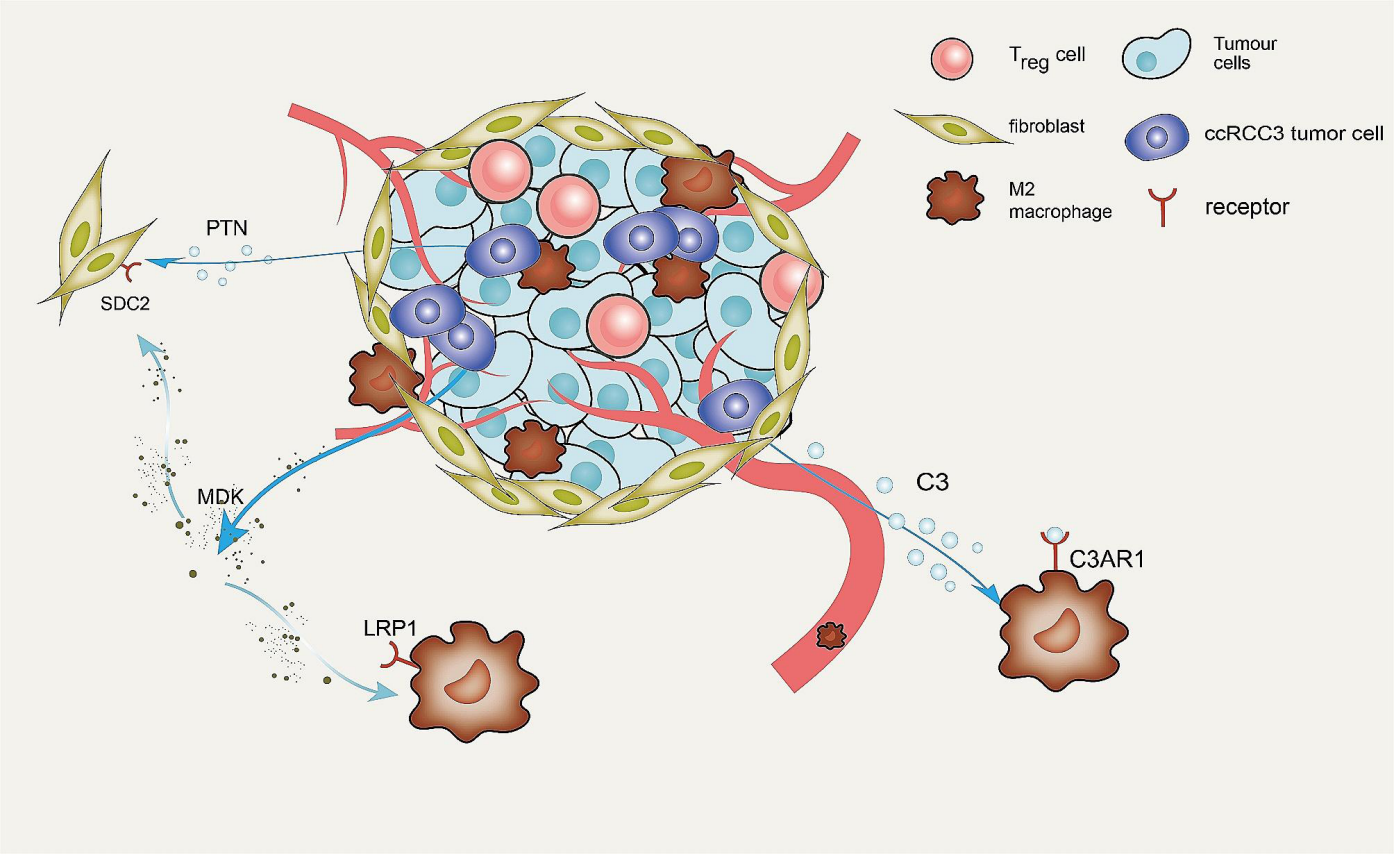
The interaction between PLOD2 + SAA1 + cancer cells and other cells in TME

The interaction between ccRCC cells and immune cells has been proven to modulate the TME and has shown significance for tumor treatments [16, 21]. The PLOD2 + SAA1 + tumor cells could communicate with other cells through ligand-receptor pairs like SPP1-CD44, MIF-(CD74 + CXCR4), and MDK-LRP1. SPP1 (secreted phosphoprotein 1) is involved in various cellular processes, including cellular signaling, angiogenesis, and immune response evasion [60, 61]. The SPP1-CD44 signaling axis has been implicated in promoting tumor progression and hampering T cell immunity. Moreover, targeting this axis has shown promise in restoring T cell function [62]. Macrophage migration inhibitory factor (MIF) has been shown to promote cancer cell proliferation and metastasis by activating multiple signaling pathways [63, 64]. Recent research has uncovered that the MIF-CD74 axis may suppress the anti-tumor immune response by attracting TAMs or directly inhibiting T cell activation [65, 66]. MDK (midkine) which is a heparin-binding growth factor that plays a role in various biological processes, including cell growth, survival, and differentiation. It is also associated with tumor progression, metastasis, angiogenesis, and resistance to chemotherapy and radiotherapy [67]. MDK secreted by tumor epithelial cells binds to its receptor LRP1 on macrophages, promoting the infiltration of immunosuppressive macrophages. It suggested that the activation of M2 macrophages through the MDK-LRP1 interaction promotes the progression of tumors [68]. Furthermore, one research has shown that increased expression of *SDC2* in CAFs (Cancer-Associated Fibroblasts) promotes tumor growth, finding the role of high *SDC2* expression in CAFs and its correlation with aggressive cancer phenotypes and poor patient survival [69], and in our study, we found PLOD2 + SAA1 + tumor cells communicated with SDC2 + CAFs through PTN-SDC2 and MDK-SDC2 pairs, which may play an important role in the development and progression of tumors. Taken together, the main crosstalk between PLOD2 + SAA1 + tumor cells and other cell types was depicted in Fig. 9. Finally, we further found that MECRGS showed promise in predicting patient outcomes after immunotherapy. We underscore the significance of comprehending the interplay between tumor cells, immune cells, and therapeutic interventions in cancer treatment.

However, there are several limitations in our study. First, our utilization of retrospective cohorts from online public databases highlights the necessity for larger prospective clinical studies involving multiple centers to confirm our findings. Secondly, conducting functional experiments is essential to validate the mechanisms of intercellular signaling in tumorigenesis. These can help identify potential biomarkers or therapeutic targets specific to ccRCC and improve our understanding of the underlying mechanisms involved in this type of cancer.

## Conclusions

Based on machine learning algorithms and various independent validation datasets, MECRGS is a major improvement compared to previous models. The excellent performance and adaptability of our signature in various datasets underscore its strength and credibility as a valuable clinical tool. Our exploration of intercellular communications involving PLOD2 + SAA1 + tumor cells provides insights into the underlying molecular mechanisms driving tumor development and metastasis. However, more research is required to fully understand the functional roles of these cells in ccRCC progression.

### Electronic supplementary material

Below is the link to the electronic supplementary material.

Supplementary Material 1

Supplementary Material 2

## Data Availability

All data used in this work can be obtained from the Gene Expression Omnibus (GEO; https://www.ncbi.nlm.nih.gov/geo/), the Cancer Genome Atlas (TCGA) datasets (https://xenabrowser.net/), and the Office of Cancer Clinical Proteomics Research (CPTAC: https://proteomics.cancer.gov/programs/cptac).
