# Supplementary material for: Comprehensive investigation of malignant epithelial cell-related genes in clear cell renal cell carcinoma: development of a prognostic signature and exploration of tumor microenvironment interactions
Source: J Transl Med. 2024 Jul 1;22:607. doi: 10.1186/s12967-024-05426-x (PMC11218120; doi:10.1186/s12967-024-05426-x)
Supplement: Supplementary file 2 — Supplementary Material 2 [file 12967_2024_5426_MOESM2_ESM.pdf]

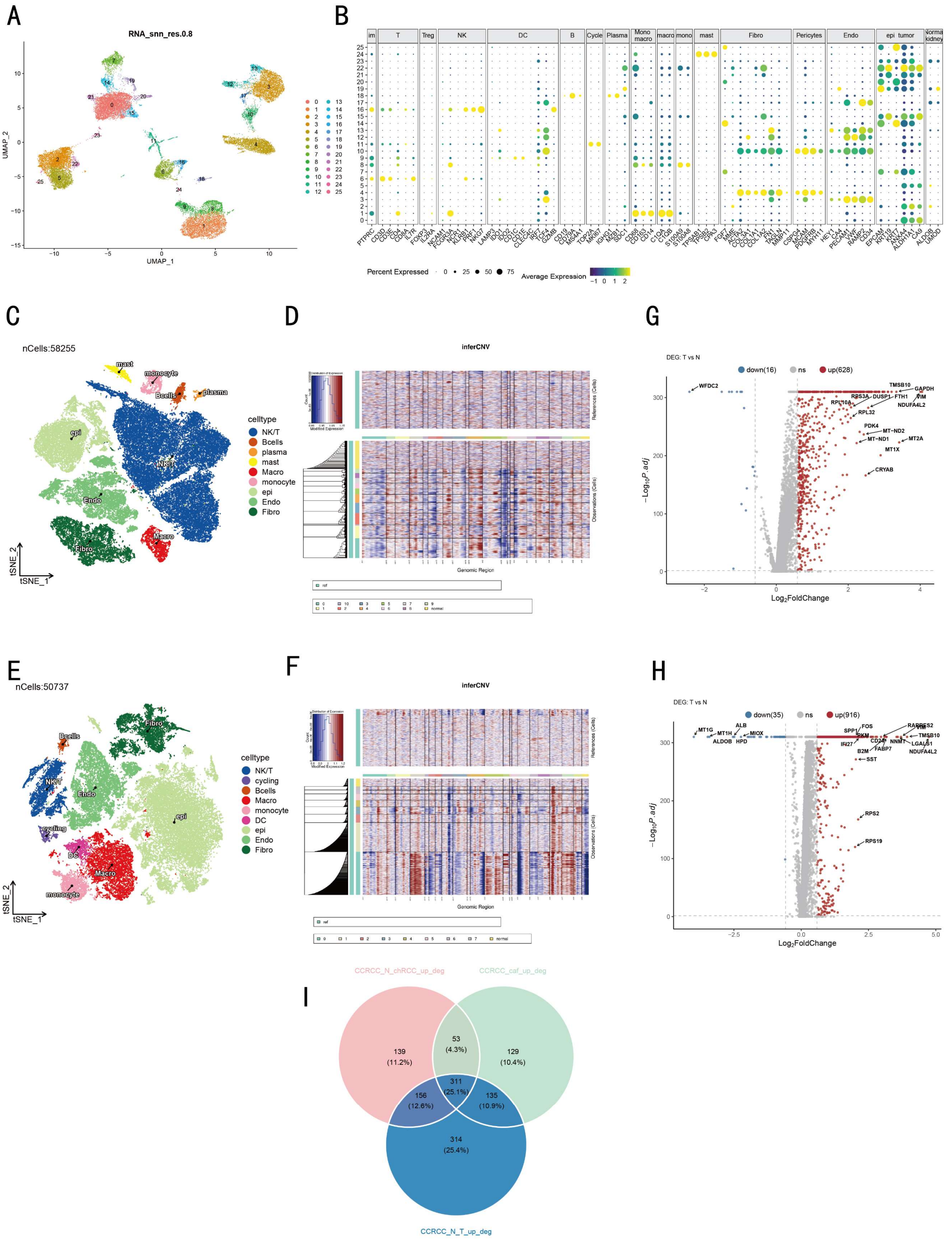

Supplementary Figure 1 Identification the DEGs between cancer cells and normal epithelial cells.

(A) Umap plot demonstrating the distribution of different cell clusters.

(B) Dot plot illustrating the distribution of marker genes across clusters. The size of each dot reflects the proportion of expressing cells, while the color coding represents the expression levels normalized by z-scores.

(C, E) t-SNE plot demonstrating the distribution of different cell types of GSE210042 (C) and GSE156632 (E).

(D, F) CNV landscape revealing differences between malignant and normal cells of GSE210042 (D) and GSE156632 (F).

(G, H) Volcano plot of DEGs between malignant and non-malignant cells of GSE210042 (G) and GSE156632 (H).

(I) Venn plot of tumor cells highly expressed genes from GSE159115, GSE210042 and GSE156632.

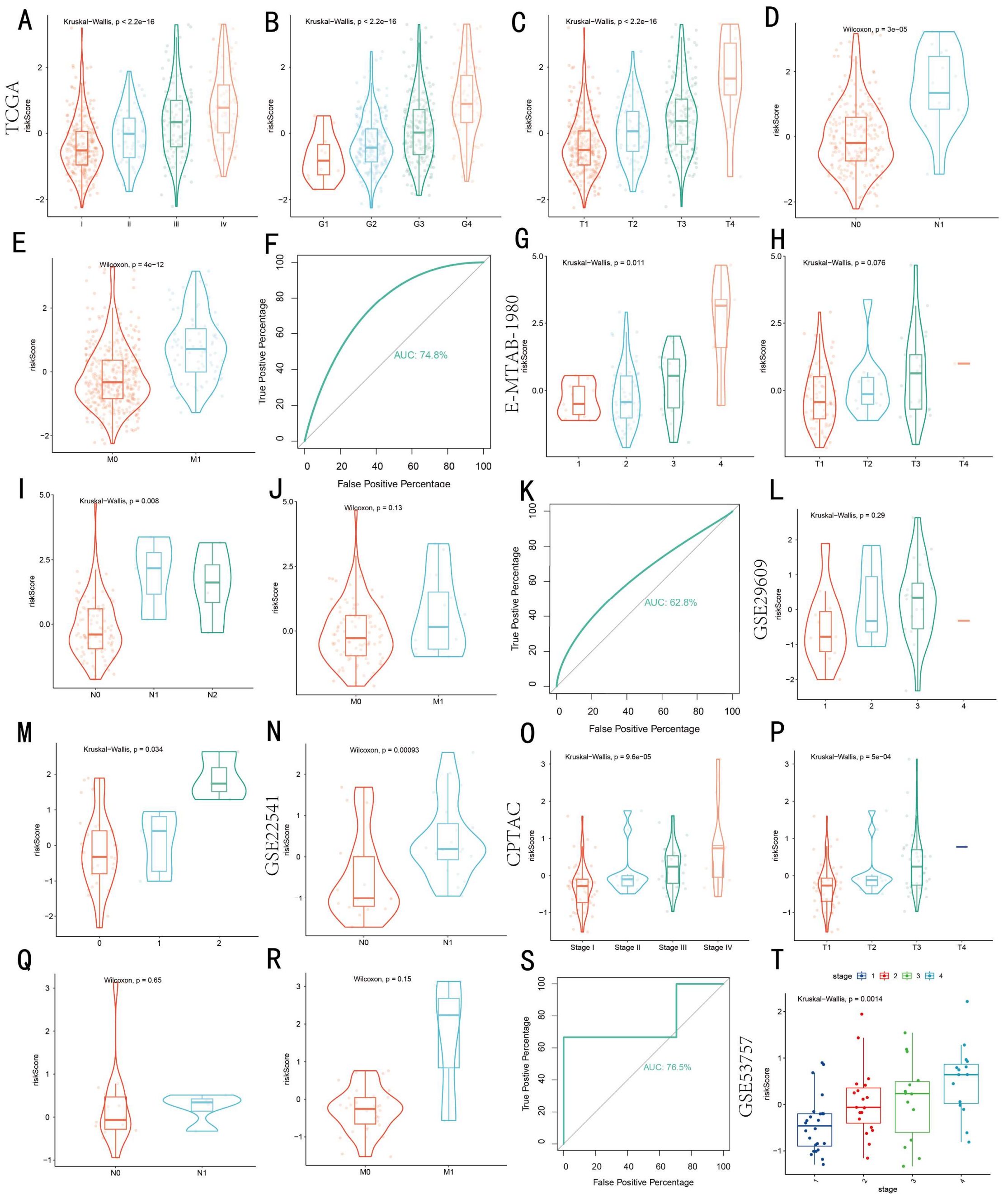

Supplementary Figure 2 Evaluation of the MECRGS model.

(A-E) Violin plots comparing the risk scores between different subgroups stratified by AJCC stage, pathological grade, T, N, and M stage in TCGA cohort.

(F) ROC curves of MECRGS in predicting the metastasis of the ccRCC in TCGA cohort.

(G-J) Violin plots comparing the risk scores between different subgroups stratified by pathological grade, T, N, and M stage in E-MTAB-1980 cohort.

(K) ROC curves of MECRGS in predicting the metastasis of the ccRCC in TCGA cohort.

(L, M) Violin plots comparing the risk scores between different subgroups stratified by T and N stage in GSE29609 cohort.

(N) Violin plots comparing the risk scores between different subgroups stratified by N stage in GSE22541 cohort.

(O-R) Violin plots comparing the risk scores between different subgroups stratified by AJCC stage, T, N, and M stage in CTPAC cohort.
(S) ROC curves of MECRGS in predicting the metastasis of the ccRCC in CTPAC cohort.
(T) Violin plots comparing the risk scores between different subgroups stratified by AJCC stage in GSE53757 cohort.

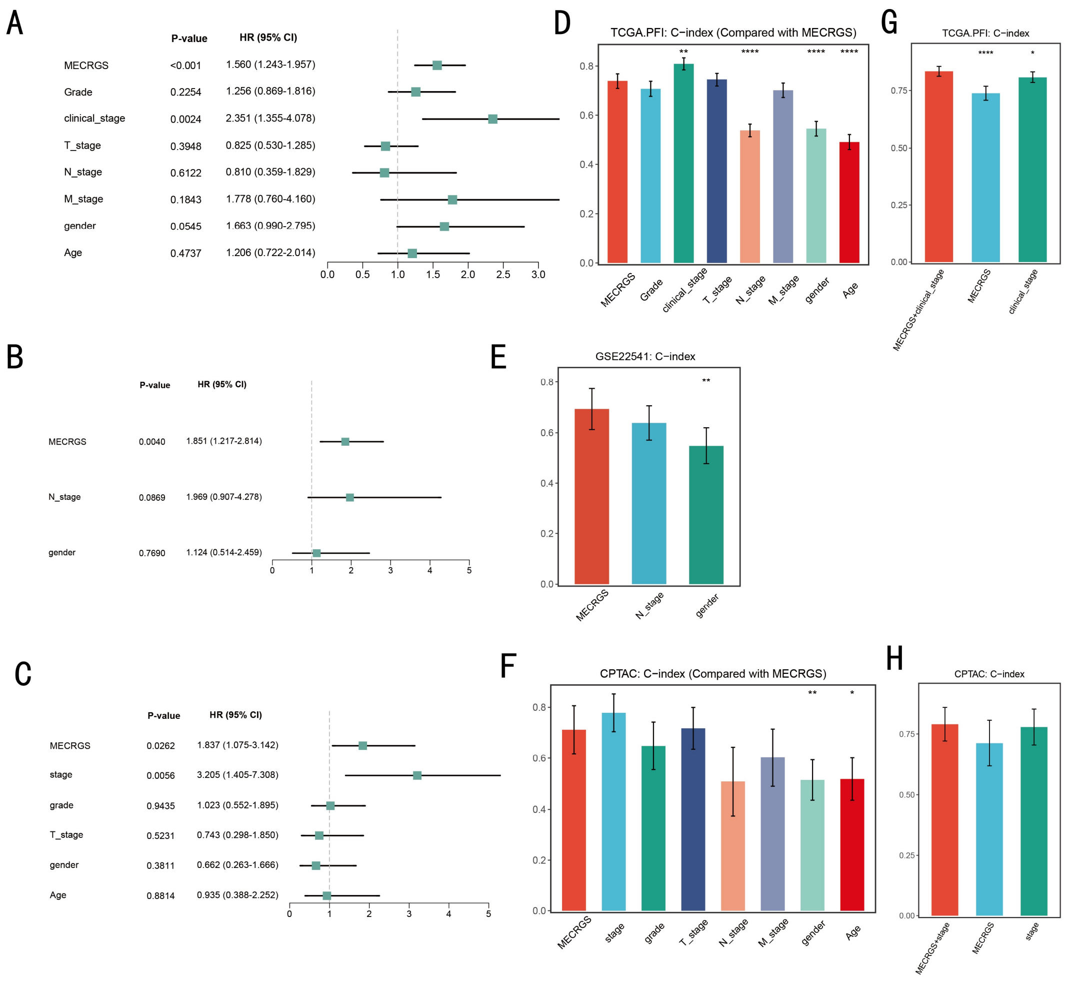

Supplementary Figure 3 The performance of MECRGS compared with clinical traits.

(A, B, C) Multivariate Cox regression of MECRGS in TCGA-KIRC, GSE22541 and CTPAC datasets.

(D, E, F) The performance of MECRGS compared with other clinical and molecular variables in predicting prognosis in TCGA-KIRC, GSE22541 and CTPAC datasets.

(G, H) MECRGS + AJCC Stage was compared with MECRGS and AJCC stage alone in predicting prognosis in TCGA-KIRC and CTPAC datasets.

Statistic tests: two-sided z-score test. Data are presented as mean ± 95% confidence interval [CI]. *P <0.05, ***P <0.001, ****P <0.0001.

A

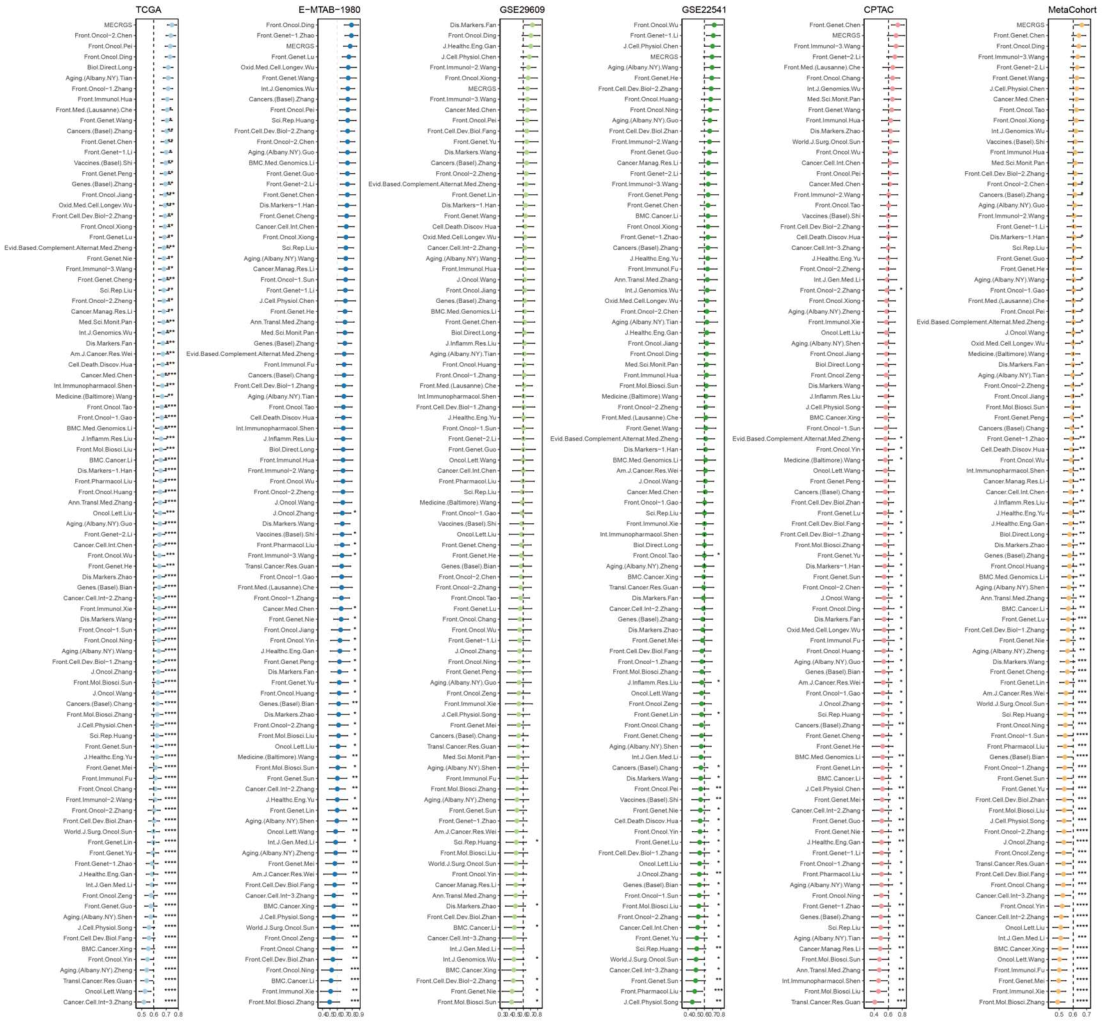

Supplementary Figure 4. Comparisons between MECRGS and 92 previously published signatures. (A) C- indexes of MECRGS and 92 published signatures in the different cohort and Meta- cohort (consisted of all cohorts). Z- score test: *p<0.05, **p<0.01, ***p<0.001, ****p<0.0001.

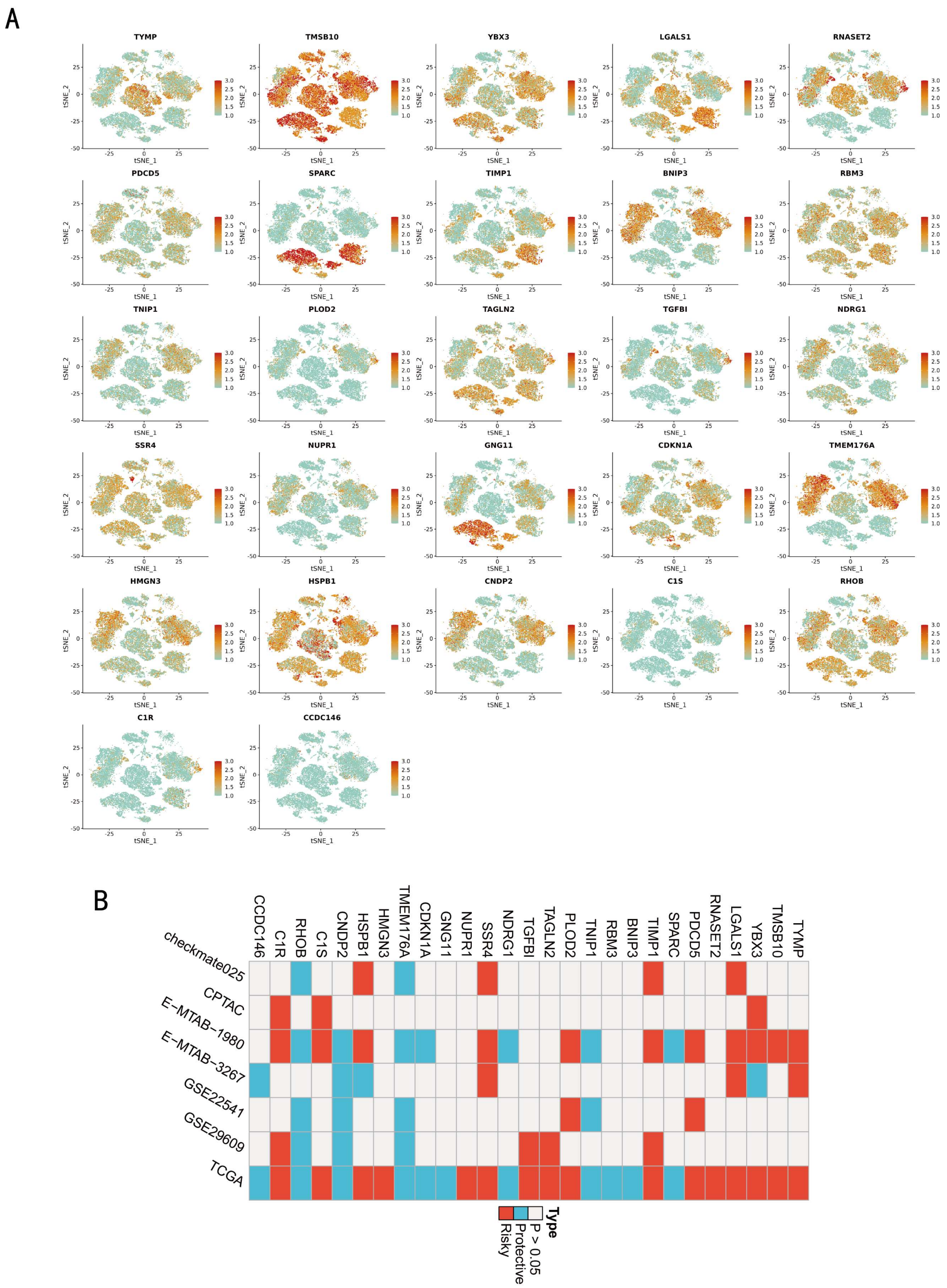

Supplementary Figure 5 Evaluation of the hub genes of MECRGS in scRNA-seq and bulk RNA levels.

(A) The expression of hub genes of MECRGS in various cell types by single-cell RNA-seq analysis.

(B) Univariate Cox regression analysis of MECRGS hub genes different datasets.

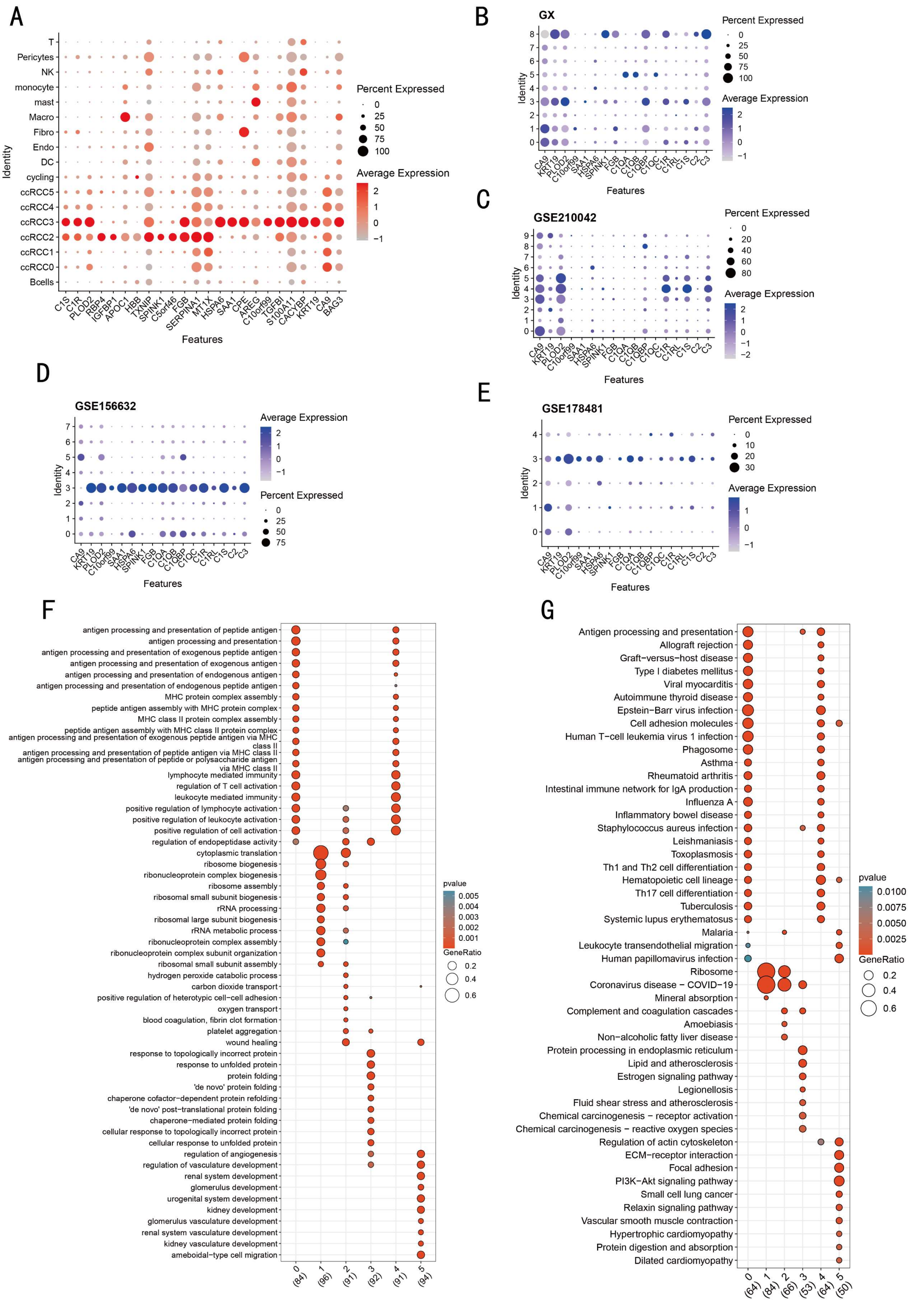

Supplementary Figure 6 Identification of different clusters of tumor cells.

(A-E) Identification of PLOD2+SAA1+tumor cells in GSE159115, GX cohort, GSE210042, GSE156632 and GSE178481 scRNA datasets.

(F, G) Dotplot showing GO and KEGG pathway analysis of six ccRCC subclusters.
